# The Problem of Molecular Target Choice for CAR-T Cells in Acute Myeloid Leukemia Therapy

**DOI:** 10.3390/ijms26125428

**Published:** 2025-06-06

**Authors:** Varvara Maiorova, Murad D. Mollaev, Polina Vikhreva, Alexey Kibardin, Michael A. Maschan, Sergey S. Larin

**Affiliations:** Dmitry Rogachev National Medical Research Center of Pediatric Hematology, Oncology and Immunology, Moscow 117198, Russia

**Keywords:** acute myeloid leukemia, CAR T, target choice

## Abstract

Recently, the chimeric antigen receptor (CAR)-T approach represented a breakthrough in the treatment of B-cell malignancies, encouraging the application of the approach for other hematological diseases, such as acute myeloid leukemia (AML). Heterogeneity and antigen variation in the pathological cell population hinder the choice of molecular targets in the case of AML. In this review, the critical aspects were described that are usually considered when selecting molecular targets for the new CAR genetic constructs. The role of AML-associated antigens in AML progression was covered. In conclusion, we proposed an approach that may allow the elimination of pathological cells in AML more effectively.

## 1. Introduction

Specific chimeric antigen receptor (CAR) T cells targeting the CD19 (cluster of differentiation 19) surface antigen have become a breakthrough in the therapy of B-ALL (B-cell acute lymphoblastic leukemia) [1]. Currently, the United States Food and Drug Administration has approved seven CAR-T therapies for the treatment of refractory or relapsed (r/r) B-cell acute lymphoblastic leukemia (Tisagenlecleucel, Obecabtagene autoleucel), r/r large B-cell lymphoma (Tisagenlecleucel, Axicabtagene Ciloleucel, Lisocabtagene Maraleucel), r/r mantle cell lymphoma (Brexucabtagene Autoleucel), and multiple myeloma (Idecabtagene Vicleucel, Ciltacabtagene Autoleucel) [2,3]. Apart from the latter two, they target CD19. Therefore, CD19 may present a successfully chosen molecular target.

CAR-T cells’ success in B-ALL treatment may be explained by the localization of blast cells in the bone marrow and peripheral blood, making them accessible for CAR-T cells. Peripheral blood is a natural site for T-lymphocyte circulation, which probably facilitates the contact between CAR-T and malignant cells. T-cells’ tissue distribution stays fragile, which is consistent with the less favorable efficacy of CAR-T cell-based therapy against solid tumors. Here, we will not discuss the issue in detail, given the published elegant reviews (e.g., [4]).

Addressing other blood disorders’ treatment based on the CAR-T approach seems quite promising, particularly acute myeloid leukemia (AML). The overall survival for adults with AML remains poor, especially in the oldest age group [5]. Therapy protocols include high-dose chemotherapy regimens that may not be effective or even applicable for patients with comorbidities and complicated somatic status [6]. In AML, transformed cells inhabit the bone marrow and peripheral blood, similarly to B-ALL. Therefore, CAR-T cell therapy may become a more effective and specific approach to treat AML.

The biology of AML differs from that of ALL. In B-ALL, it was possible to find unique antigens that are present on the surface of all blast cells [7]. The AML pathologic population is heterogeneous, with clones of blast cells that differ both genotypically and immunophenotypically [8]. In this review, we examine the criteria for selecting a molecular target for CAR-T cells and analyze the known AML antigens based on these criteria.

## 2. Choosing the Molecular Target for CAR-T Cells

Based on the CD19-specific CAR-T therapy success, there is a consensus regarding the criteria for promising molecular targets [9]. Tumor-associated antigens should be (1) presented on the surface of all tumor cells, to effectively eliminate the malignant cell population and (2) tumor-specific to avoid serious side effects related to “on-target off-tumor” toxicity.

The ideal tumor-specific antigens are virtually unknown. CD19 is present on the surface of all cells within the B-cell lineage of hematopoiesis, including healthy cells [10]. Targeting CD19 leads to prolonged B-cell aplasia, which is a significant side effect [11]. The same holds true for the B-cell markers CD20 [12] and BCMA (B-cell maturation antigen) [13]. Fortunately, the patients’ plasma cell functions may be compensated through regular injections of intravenous immunoglobulin preparations [3]. Thus, a more precisely formulated criterion for the tumor specificity can be stated as follows: (2b) it should be possible to maintain a satisfactory patient health status, taking into account the side effects of targeting the tumor-associated antigen.

Also, it cannot be ignored that malignant cells are able to adapt to therapy [14]. In the case of anti-CD19 CAR-T cell therapy, relapse may develop with the loss of CD19 [15]. The specific targeting of CD19 becomes ineffective, necessitating alternative immunotherapeutic agents that are specific to other antigens. Then, the design of the new CAR coding genetic construct should include considering the potential for antigen-negative relapse. Based on this, targeting the antigens that bear an important role in AML progression could be promising. Then, loss of the antigen would be accompanied by loss of the benefits that were derived from the antigen functioning in blast cells. So, target molecule (3) should bear a crucial biological function, providing malignant cells with survival, expansion, or migration benefits. Thus, in the case of antigen-negative relapse, the tumor may become less aggressive and develop at a slower rate.

## 3. Targeted Immunotherapy Issues in Acute Myeloid Leukemia

The blast cells in AML originate from normal cells of the myeloid lineage of hematopoiesis. The wide range of blood cell subpopulations belongs to the myeloid lineage of hematopoiesis. Genetic abnormalities may cause malignant transformation of a cell of a particular myeloid subpopulation, such as monocytic or granulocytic. Then, blast cells derived from various lineages exhibit distinct immunophenotypes [16]. For instance, the immunophenotype of blasts in acute monocytic leukemia would possess markers that are associated with the monocytic lineage of hematopoiesis [17]. This leads to a broad spectrum of AML types, categorized based on the origin of the cell harboring the initial genetic defect [16].

**Figure 1:**
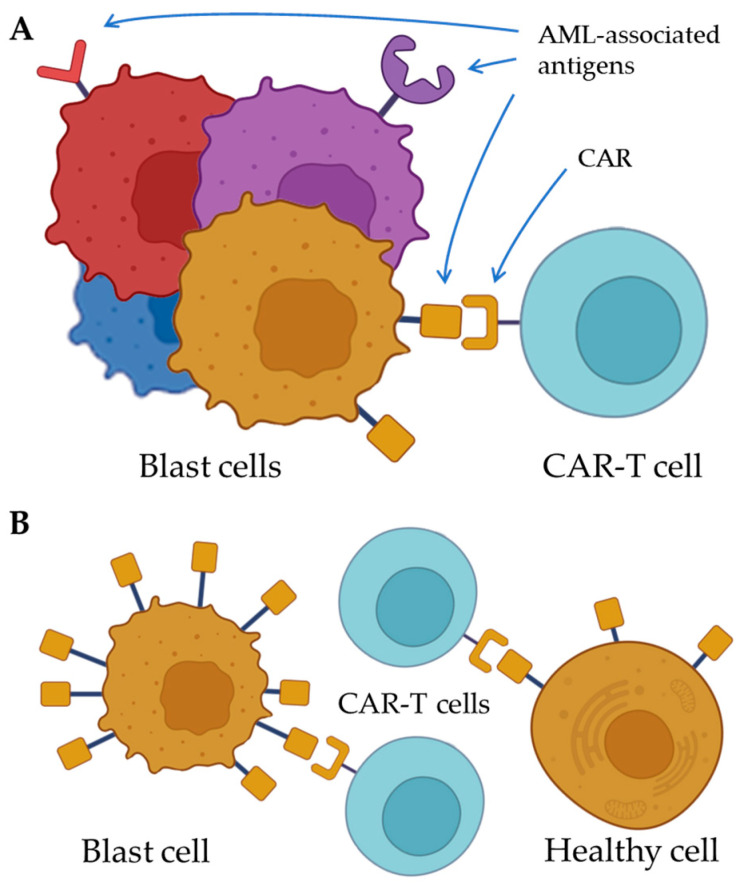
Challenges of molecular target choice for CAR-T cells in AML. (**A**) Heterogeneity of blast cells limits the effectiveness of CAR-T cell therapy of AML. (**B**) The AML-associated antigens are often expressed by the healthy cells, leading to CAR-T-mediated on-target off-tumor toxicity. CAR; chimeric antigen receptor. AML; acute myeloid leukemia.

Moreover, the blast cells of individual patients diagnosed with a specific type of AML (e.g., acute megakaryoblastic leukemia) can be significantly heterogeneous both at the genotype and immunophenotype levels (Figure 1A) [18]. Such genomic variability and diversity in biochemical and functional properties among individual AML cells enhance the ability of tumor cells to adapt to therapy or change during AML progression.

In AML, normal cellular antigens tend to be excessively present on the blast cells’ surface but also synthesized by healthy cells (Figure 1B) [19]. In vivo studies and clinical trials of CAR-T cells specific to various AML markers showed side effects such as monocytopenia, neutropenia, and bone marrow ablation due to the ‘on-target off-tumor’ toxicity [20,21,22]. The prolonged duration of the conditions would significantly impact the quality of life and may lead to lethal outcomes due to infectious or septic complications or systemic hematopoietic disturbances.

Recently, CAR-T cells have been tested not as monotherapy but as a combination with traditional therapeutics such as fludarabine/cyclophosphamide (e.g., [23,24]). This approach may ensure more thorough conditioning of the patient’s bone marrow and even serve as a preparatory stage, a ‘bridge’, to a subsequent hematopoietic stem cell transplantation (HSCT). Potentially, the cytostatic agent’s dosage might be reduced while using them in addition to CAR-T cells. Also, systemic side effects arising from prolonged elimination of blood cells may be avoided in case of further HSCT. The short-term use of CAR-T cells would also streamline the choice of molecular targets, as “on-target off-tumor” toxicity would become less relevant, expanding the variety of promising AML-associated molecular targets.

Summarizing, we emphasize that a unique AML-specific antigen that can be targeted in all cases of AML has not yet been described. We assume that the trajectory for the CAR-T cell therapy for AML differs from that for the B-cell lineage pathologies. We propose that the simultaneous targeting of a set of markers with a cocktail of CAR-Ts, specific to a variety of antigens, would allow a more accurate elimination of the heterogeneous blast population in AML.

## 4. Promising Molecular Targets for Acute Myeloid Leukemia

Cells originating from all the types of myeloid lineage may undergo tumor transformation, affecting the blast cells’ immunophenotype in AML. It is not surprising that over a dozen surface cellular markers are considered promising molecular targets for immunotherapy. The most significant ones are discussed below.

### 4.1. T-Lymphocyte Antigen—CD7

T-lymphocyte antigen CD7 is one of the earliest T-cell markers [25] and is essential for T- and B-cell interactions during lymphoid cell development [26]. The CD7 marker appears on the cell surface at the stem cell stage and remains present until the T cell is fully mature. Additionally, the CD7 marker is characteristic of natural killer (NK) cells [27].

In a mouse model with a knockout of the *CD7* gene, it was shown that the development of the mouse immune system did not depend on the presence of CD7 molecules [28]. However, CD7 protein influenced the migration of normal T cells in both in vitro and in vivo models. CD7-positive tumor cell lines exhibited similar properties as follows: Blocking the CD7 protein expression in CD7-positive cell lines reduced their metastatic capacity in the mouse model [28].

Blast cells were CD7-positive in 30% of adult acute myeloid leukemia cases [29]. The presence of this marker was associated with a more aggressive disease and resistance to chemotherapy [30,31].

Anti-CD7 CAR-T cells exhibited fratricide, which rendered CAR-T cell expansion procedures impossible. To obtain anti-CD7 CAR-T cells, the authors had to inhibit endogenous CD7 in T cells [32]. Preclinical studies have been published by D. Gomes-Silva et al. [33]. The Phase II clinical study has been completed in China recently, with 14 patients enrolled. Although no results have been posted yet [34].

### 4.2. Sialic Acid-Binding Immunoglobulin, Siglec-3—CD33

Sialic acid-binding immunoglobulin, Siglec-3, is primarily present on the myeloid cell surface, including myeloid progenitor cells [35]. Additionally, this marker is synthesized by neutrophils, NK cells, B cells, and Kupffer cells in the liver [36].

The transmembrane protein Siglec-3 carries an intracellular inhibitory ITIM (immunoreceptor tyrosine-based inhibition motif) domain [35]. By recognizing its ligand—sialic acid—on the surface of normal cells, the Siglec-3 protein initiated intracellular signaling pathways that inhibited immune cell activation [37]. Tumor cells typically expressed ligands for the Siglec-3 protein on their surface, which may contribute to the inhibitory tumor microenvironment forming [37].

Blast cells are CD33-positive in 85–90% of AML cases. Leukemic stem cells were also declared to be CD33-positive [38], making CD33 an attractive molecular target. However, the analysis of RNA sequencing and qPCR of the AML bone marrow samples revealed the presence of four splice variants of the CD33 protein in 93% of the samples [39]. According to the UniProt database, among these, two truncated variants did not contain amino acid residues that are associated with the ITIM inhibitory motifs. Therefore, despite CD33 being recognized as a promising molecular target in AML therapy, scientific knowledge about its functions in acute myeloid leukemia remains incomplete.

In the clinical study focused on AML therapy with anti-CD33 CAR-T cells, significant side effects were detected, including cytokine storm, neurotoxicity, tumor lysis syndrome, and third-degree respiratory distress syndrome [36]. Interestingly, hepatotoxicity was not observed despite the CD33 expression by Kupffer cells. Thereby one can propose the lack of CAR-T cell tissue infiltration.

### 4.3. ADP-Ribosyl Cyclase—CD38

The CD38 marker is expressed mainly by plasma cells, but it is also synthesized at various stages of differentiation by other blood cells [40] and solid organs, such as the brain, eye, prostate, stomach, pancreas, muscles, bones, and kidneys [41].

The CD38 protein is known to perform two independent functions; it may act both as a protein receptor that is involved in the inflammatory response and the recruitment of immune cells to the site of damage and as an enzyme that catalyzes the synthesis of cyclic ADP-ribose and NAADP [42]. The enzymatic functions of CD38 were accompanied by an increase in intracellular Ca^2+^ concentration, actin polarization, and consequently, enhanced migration of pathological cells in vitro [42].

In a clinical study performed by Cui, Q. et al., patients with refractory and relapsed AML who had undergone allogeneic hematopoietic stem cell transplantation were administered anti-CD38 CAR-T cells. Four out of six participants achieved remission; however, half of them relapsed within the next six months [43]. An early Phase I clinical study focusing on dual targeting of CD38 and C-type lectin-like molecule-1 (CLL-1) in AML has been terminated recently, with 3 patients enrolled. No results have been published yet [44].

Conceivably, using CAR-T cells during the conditioning phase before hematopoietic stem cell transplantation may be more effective in preventing relapses. CAR-T cells could eliminate malignant cells more thoroughly, and then healthy donor cells would restore hematopoiesis. Thus, CAR-T could become a part of combination therapy, performing its cytotoxic function while simultaneously preparing a niche for the subsequent HSCT.

### 4.4. Cell Adhesion Molecule—CD44v6

The CD44 adhesion receptor is present on the surface of many cell types. Its ligands include hyaluronan, osteopontin, fibronectin, and selectin, whose functions relate to cellular migration [45]. At least 10 variants of CD44 have been described, differing in ligands and functions [1].

The group led by Liqing Jin demonstrated in vivo that CD44 played a crucial role in the engraftment of leukemic stem cells [46]. A series of peripheral blood samples from patients with AML were injected into NOD/SCID mice. The blast cell engraftment in the bone marrow was awaited and treated with infusions of H90 antibodies specific to all the known CD44 variants. As a result, an increase in the late differentiation markers (CD14 and CD15) present on the human cells was observed compared to the immunophenotype of the initial population of blast cells. Conversely, the cells lost their ability to accumulate in the bone marrow and spleen of mice [46].

The CD44v6 splice variant is considered a tumor-associated antigen, despite being present on normal T-cells, monocytes, and keratinocytes [45]. Known ligands for CD44v6 include hepatocyte growth factor, vascular endothelial growth factor, and osteopontin, which initiate proliferative and anti-apoptotic intracellular signaling pathways.

CD44v6 is present on the surface of blast cells in 60% of AML cases [20]. The group led by Ling Tang connected increased expression of CD44v6 on the blasts’ surface in AML with mutations in the *FLT3* and *DNMT3A* genes [47].

The CD44v6 marker was used as a molecular target in a clinical study in Italy. The trial has been terminated based on the low patient recruitment rate and the diffusion of the COVID-19 emergency [48]. AML patients had not been recruited before the study was terminated. Despite the lack of clinical data regarding the efficacy of anti-CD44v6 CAR-T therapy, we consider the research important to mention, as the targeting of AML-associated isoforms of surface markers may represent a promising approach to reducing on-target off-tumor toxicity.

### 4.5. Ligand of the Tumor Necrosis Factor Receptor—CD70

The increased synthesis of the CD70 ligand for the tumor necrosis factor receptor CD27 can be stimulated by the activation of the immune cells, namely, lymphocytes and dendritic cells [49].

The interaction between CD27 and CD70 supported the stem cell properties of blast cells in AML [50]. Blocking antibodies specific to CD70 reduced the expression of stemness-associated genes and initiated differentiation of blast cells in vivo. The presence of the CD70 on the surface of the blast cells was associated with poor prognosis in AML.

During preclinical studies, anti-CD70 CAR-T cells successfully eliminated AML blast cells and leukemic stem cells but not hematopoietic stem cells [51]. Clinical studies referred to r/r AML treatment recruit patients. Interestingly, both anti-CD70 CAR-T cells [52] and anti-CD70 NK cells that have been additionally transduced with Interleukin-15 (IL-15) [53] are being studied in clinics. The results regarding the efficacy of anti-CD70 NK cells are of particular interest, as patients should receive an allogeneic cell product rather than an autologous one, which is the traditional option relevant to CAR-T.

### 4.6. Leukocyte Immunoglobulin-like Receptor B ILT3—CD85k

Leukocyte immunoglobulin-like receptor B4 (LILRB4, or ILT3) is normally synthesized by dendritic cells, monocytes [54], endothelial cells [55], and osteoclasts [56].

The intracellular portion of ILT3 contains inhibitory ITIM domains [54], which were important for inhibiting T-cell activation in AML and the infiltration of leukemic cells [57]. The authors constructed chimeric proteins containing the extracellular domain of LILRB4 and the intracellular domain of LILRB1 and vice versa. The intracellular domain of LILRB4, but not that of LILRB1, mediated T-cell suppression and AML cell migration [58]. Thus, the increased synthesis of ILT3 protein in AML may be part of the tumor’s mechanism for evading immune surveillance.

In AML, ILT3 was predominantly expressed in the blast cells of patients with M4 and M5 AML types [59]. A sample was considered positive for the ILT3 marker if more than 10% of the cells were stained with the specific antibodies.

Currently, the clinical study is recruiting patients to investigate the efficacy of anti-ILT3 CAR-T cells in relapsed or refractory AML of M4 and M5 types [60].

### 4.7. Alpha Chain of the Granulocyte-Macrophage Colony-Stimulating Factor Receptor Complex GM-CSFR—CD116

The alpha chain of the granulocyte-macrophage colony-stimulating factor receptor (GM-CSFRα, also known as CD116) is a marker of early precursors in the myeloid lineage of hematopoiesis [61]. Its ligand GM-CSF is known to stimulate proliferation and differentiation [62].

GM-CSFRα is overrepresented on the surface of blast cells in 63–78% of AML cases [63,64], particularly in association with mutations in the *FLT3* gene [65]. The pathological cells’ populations in patients exhibited heterogeneity regarding the CD116 marker [66]. The highest percentage of cells expressing the antigen was found in the acute myelomonocytic leukemia (M4 type) and acute monocytic leukemia (M5 type) samples. In those cases, 60% and 70% of all blast cells, respectively, were CD116-positive.

In a mouse model, anti-GM-CSFR CAR-T cells demonstrated an anti-tumor effect [66].

### 4.8. Stem Cell Factor Receptor c-Kit—CD117

The stem cell factor receptor c-Kit, also known as CD117, plays a critical role in the homeostasis of hematopoietic stem cells [67]. It is normally present on hematopoietic stem cells and mast cells [68]. Activation of the c-Kit receptor is known to trigger the intracellular signaling pathways that ensure cell survival and proliferation [68].

Blast cells present CD117 in 80–90% of AML cases, and the antigen is associated with a poor prognosis [69]. The blast cell population is often heterogeneous as follows: When staining bone marrow samples with anti-CD117 antibodies, 55–80% of blasts were found to be positive [70]. Interestingly, the density of the c-Kit on the surface of the bone marrow blast cells correlated with a lower number of blast cells in AML patients [71]. The intensity of cell fluorescence in the samples from patients with a high tumor burden was significantly lower than that in the cells from patients with a low tumor burden.

In a mouse model, anti-CD117 CAR-T cells completely eliminated both healthy bone marrow and malignant cells [72]. To limit side toxicities, anti-CD117 CAR-T cells were eliminated in vivo by administering standard doses of anti-thymocyte globulin (ATG). Based on that, targeting the CD117 marker in AML is likely promising, although its application may find its place as a preparatory conditioning stage before HSCT. Efforts in this area are presently at the preclinical research stage.

### 4.9. Alpha Chain of the IL-3 Receptor Complex—CD123

The alpha chain of the IL-3 receptor complex (CD123) is normally synthesized predominantly by myeloid cells [73]. Activation of the IL-3 receptor is known to initiate intracellular signaling pathways that promote cell survival and proliferation [74].

CD123 marker was detected on blast cells in 70–80% of AML cases, and the presence of CD123 was associated with refractoriness [75]. In 55% of AML cases, more than 80% of blast cells were stained positive with anti-CD123 antibodies [76].

Given the prevalence of the CD123 marker on the surface of healthy cells, it is not surprising that anti-CD123 CAR-T cells eliminated not only malignant cells but also healthy myeloid precursor cells [77]. This contributes to significant toxic side effects associated with the investigated anti-CD123 CAR-T cells. The Phase I clinical study was completed in China in 2010 with 10 patients enrolled. No results have been posted [78].

### 4.10. Tyrosine Kinase Receptor Flt3—CD135

Flt3 receptor is primarily a marker of early hematopoietic progenitor cells and dendritic cells [79]. Flt3-ligand binding is known to stimulate the activation of intracellular signaling pathways, resulting in cell proliferation.

Flt3 is present in increased amounts on the blast cell surface in more than 80% of AML cases [80]. Approximately 30% of AML cases with blasts carry an activating mutation in the *FLT3* gene sequence, known as Flt3-ITD, which enhances blast proliferation and is associated with poor prognosis [79].

Currently, the Flt3 antigen is featured as a molecular target for CAR-T therapy in AML in the four clinical studies [81,82,83,84]. Anti-Flt3 CAR-T cells are being tested both as monotherapy for AML and in combination with chemotherapeutic agents fludarabine and cyclophosphamide.

### 4.11. NK Cell Ligand B7-H3—CD276

The protein B7-H3 (CD276) belongs to the B7 protein family, which also includes the immune checkpoint inhibitor PD-L1 (B7-H1). Unlike PD-L1, the B7-H3 protein was able to perform both inhibitory and stimulatory functions, regulating the immune cells’ activity [85]. In normal tissues, B7-H3 coding mRNA was present in the cells of many organs and tissues, although the protein synthesis level was low. The synthesis of B7-H3 in T, B, and NK cells can be stimulated by inflammatory cytokines or phorbol myristate acetate and ionomycin [85].

At least two isoforms of B7-H3 have been described, differing in their extracellular parts as follows: One variant contained two immunoglobulin-like domains, while the other contained four, with the latter form being predominant [85].

The B7-H3 protein was shown to perform various functions during AML, starting from regulating blast cells’ proliferation and their migration to participating in the inhibitory tumor microenvironment formation, reducing the immune cells’ activity, particularly NK cells [85]. Increased expression of B7-H3 on the blast cells’ surface in AML correlated with decreased overall survival of patients [86].

AML patients’ samples were positive for the B7-H3 marker in 37% of cases [87]. Analysis of the transcriptome of bone marrow cells from AML patients revealed significant differences in the level of B7-H3 mRNA expression between adults (58%) and children (22%) [88]. In this context, monocytic AML was highlighted in the B7-H3 overexpression in 40–60% of bone marrow samples [89].

Testing of anti-B7-H3-CAR-T cells in AML therapy is currently in the preclinical research stage [90,91].

### 4.12. Inhibitory Protein Siglec-6—CD327

Inhibitory protein Siglec-6 is present on the surface of B cells, mast cells, and placental cells [92]. Like Siglec-3 (marker CD33), the intracellular part of the Siglec-6 protein was shown to contain ITIM suppressor domains. The group led by H. Jetani used the Siglec-6 marker as a target molecule for targeting AML blast cells [92]. The marker showed that AML patient blast cells were heterogeneous.

The Siglec-6 antigen was used as a molecular target in preclinical studies focused on the therapy of leukemia and lymphoma. In mouse models, remission with preserved hematopoietic stem cells has been demonstrated [92]. Siglec-6 could be an interesting target molecule due to its limited expression on healthy cells; however, the biological functions of this marker are not yet fully understood. A Phase I/II clinical study is recruiting patients in China [93].

### 4.13. The Protein Containing Immunoglobulin and Mucin Domains, TIM-3—CD366

The TIM-3 protein is known to participate in regulating the inflammatory response, specifically inhibiting macrophages, Th1, and Th17 cells [94]. TIM-3 inhibited the cytotoxic function of T cells and participated in the inhibitory tumor microenvironment in AML [95].

The TIM-3 antigen was detected on the surface of 6% of blast cells in the AML samples, but the presence of TIM-3 correlated with the patient’s belonging to the high-risk AML group [96].

Anti-TIM-3 CAR-T therapy was tested on in vitro and in vivo models [97]. A Phase I/II clinical study is recruiting patients in China to target both CD123 and TIM-3 antigens in relapsed/refractory AML [98].

### 4.14. Lectin-like C-Type Domain CLL-1 Protein

Lectin-like C-type domain protein belongs to the family of C-type lectin-like domains that recognize molecular patterns associated with damage or pathogens and regulate innate and adaptive immunity. The intracellular part of CLL-1 contains a sequence that was similar in function to the inhibitory ITIM domain, as well as a domain with currently unknown functions [99].

Human CLL-1 is predominantly synthesized by cells of the hematopoietic lineage: it is present on mature granulocytes and monocytes, as well as in ~60% of their precursor cells, 40% of progenitor cells, and 2.5% of hematopoietic stem cells (CD34+ CD38−). It is believed that CLL-1 plays an inhibitory role in regulating the activity of granulocytes and monocytes. Ligands for CLL-1 are not yet sufficiently studied. CLL-1 was not synthesized by T-, B-, or NK cells, erythrocytes, or their precursors [100].

Blast cells were CLL-1-positive in more than 80% of AML cases [101]. The antigen was also found on leukemia stem cells, which tend to be drug-resistant [99]. However, bone marrow samples of the AML patients were revealed to be significantly heterogeneous regarding the CLL-1 marker [100]. Moreover, overall survival rates for patients with varying levels of CLL-1 expression showed considerable differences, even within the high-risk cohort. A high expression level of CLL-1 correlated with better overall survival [102].

The first patient who was administered anti-CLL-1 CAR-T cells remained in remission for over 10 months [103], which is an impressive result considering the aggressive nature of acute myeloid leukemia. Several clinical studies focusing on anti-CLL-1 CAR-T therapy are currently active and recruiting patients (e.g., [104]). The Phase I study was terminated in 2024 due to futility [105].

### 4.15. Folate Receptor FRβ

The folate receptor (FR) family consists of four protein receptors that bind folic acid and differ in their tissue distribution. The FRβ protein is predominantly synthesized by hematopoietic cells and is present in the spleen, thymus, and placenta, stimulating leukocyte growth [106]. Notably, FRβ protein is present on the surface of human monocytes and macrophages, according to staining results with FRβ-specific antibodies [107]. Interestingly, staining those cells with fluorescently labeled natural ligand of FRβ, folic acid, demonstrated the presence of a functional FRβ receptor on monocytes but not on macrophages.

The myeloid antigen FRβ is overrepresented in 70% of AML cases [108]. Anti-FRβ CAR-T cells have exhibited cytotoxic effects in vitro and in vivo [108,109]. To date, anti-FRβ CAR-T cells have not been administered to humans.

### 4.16. NKG2D Receptor Ligands

The NKG2D receptor is an activating receptor synthesized by NK cells, γδ T-cells, CD8-positive T-cells, and certain subtypes of CD4-positive T-cells [110]. The NKG2D protein is important for recognizing and eliminating damaged, infected, or transformed cells. The natural ligands for the human NKG2D receptor are the major histocompatibility complex class I polypeptide–related sequence A and B proteins (MICA and MICB) and UL16 binding proteins (ULBP1–ULBP6). The synthesis of NKG2D ligands can be activated by cellular stress or abnormalities characteristic of tumor transformation. NK cells identified the damaged/pathological cells and eliminated them via the NKG2D receptor and its ligands on the surface of other cells interaction [110].

More than a hundred variants of MICA, forty variants of MICB, and over a dozen variants of ULBP proteins have been described [110]. Given the high variability of NKG2D ligands, it is logical to use the extracellular receptor fragment of NKG2D as the recognizing part of the chimeric antigen receptor instead of the scFv fragment of antibodies. The ScFv-fragment would be specific to one molecular target, while it would be possible to target the whole family of NKG2D ligands using the NKG2D fragment as the recognizing domain of CAR.

At least one of the NKG2D ligands was present on the blast cells’ surface of the majority of AML patients (75%) in the comprehensive study of the mechanisms to evade NKG2D-mediated immunosurveillance during leukemia [111].

The Phase I clinical trial was completed in the United States in 2018, with 12 patients enrolled [112]. No results have been posted. Currently, several clinical studies are focusing on NKG2D-CAR-T therapy and NKG2D-CAR NK therapy [113,114,115]. Interestingly, various therapeutic agents increased the synthesis of NKG2D ligands in AML, such as idarubicin and tretinoin [116]. Using targeted therapy specific to NKG2D ligands, in combination with agents that induce hyperexpression of the NKG2D ligands on the pathological cells’ surface, represents a potentially interesting approach.

## 5. Conclusions and Perspectives

Currently, there are no CAR-T products approved for the treatment of acute myeloid leukemia. The most promising outcomes in AML therapy are observed following hematopoietic stem cell transplantation [117]. All registered CAR-T therapies are designed for the treatment of B-cell lymphoid hematologic malignancies [2]. However, numerous other tumor types exist for which effective therapies have yet to be developed. These diseases present new challenges for the CAR-T approach.

The development of new CAR-T products for the treatment of any condition necessitates thorough literature analysis and the selection of a molecular target. The choice of molecular target is largely determined by the biology of the specific disease. Generally, within neoplasms, two distinct classes can be highlighted as follows: hematologic malignancies and solid tumors. The aforementioned B-cell lymphoid hematologic malignancies and acute myeloid leukemia fall within the category of hematologic pathologies. Currently, addressing solid tumor therapy is an inspiring challenge for CAR-T research. The primary issues relating to the CAR-T approach in the treatment of solid tumors today are not merely the selection of a molecular target but also the low infiltration of CAR-T cells into the tumor. The challenges associated with the CAR-T therapy of solid tumors have been extensively reviewed in numerous articles (see e.g., [4]). In our review, we focused specifically on hematologic pathologies, particularly acute myeloid leukemia.

The problem of target choice for CAR-T therapy in AML is complicated by the heterogeneity of the immune phenotype of the pathological cell population. The analysis of data on the well-known molecular targets revealed that none can be considered an AML-specific antigen, as they are expressed by only a fraction of malignant cells (Table 1). Moreover, most of the potential targets for CAR-T are also synthesized by healthy cells, which raises concerns about significant on-target off-tumor toxicity. Then, the choice of a single target or the application of monotargeted CAR-T therapy over a prolonged period appears to be an ineffective approach in treating AML.

**Figure 2:**
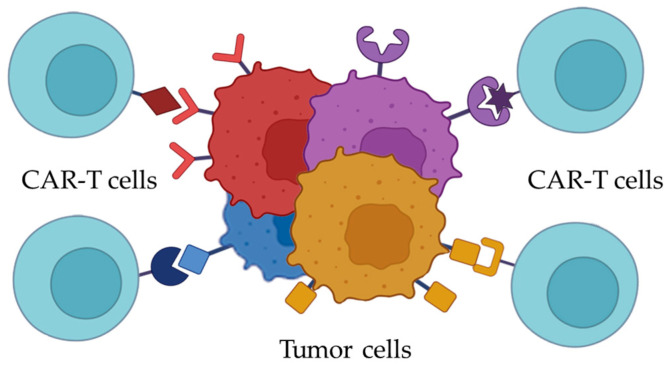
Heterogenic blast population in AML may be targeted with a cocktail of CAR-Ts, each specific to an AML-associated antigen expressed by a pathological subpopulation.

In the context of AML, the aim of selecting a molecular target for CAR-T may need to be reconsidered as the identification of multiple molecular targets. Based on immunophenotyping results of the samples from particular patients, a panel of surface antigens could potentially be selected to target 100% of the malignant cells, including minor pathologic populations (Figure 2). Multitargeting CAR-T is also being studied nowadays. For instance, in a recent study by Haubner et al., T-cells were modified with a construct encoding two chimeric antigen receptors, each specific to a different marker. Ideally, a range of genetic constructs encoding CARs targeting either the most frequently encountered or correlating with poor prognosis AML-associated markers should be available. This would allow for the selection of a CAR combination that would be both necessary and sufficient for effective treatment of each specific AML case.

Multitargeting, however, complicates the issue of potential on-target off-tumor toxicity. The idea of using a combination of CAR-T products necessitates consideration of the likely toxicity posed by each CAR within the set. To mitigate the severity and duration of adverse effects, it might be of potential interest to use CAR-T as a short-term therapy during the preparatory phase for HSCT. This approach could allow for more specific and effective preparation of the niche for healthy donor cells, thereby reducing the need for high doses of chemotherapeutic agents typically employed prior to HSCT.

Summarizing, we have analyzed the blast cells’ antigens within the context of the ideal molecular target, discussed at the beginning of the review (Table 1). Given the diversity and heterogeneity of AML-associated antigens, it is unlikely to eliminate 100% of blast cells with a CAR-T cell therapeutic product specific to one AML marker. We can conclude that introducing a set/cocktail of CAR-T cells specific to various AML antigens could be a promising direction. Then, it would be feasible to tailor a combination of specific CAR-T cell variants that would best suit the patient based on the results of blast cells’ immunophenotyping. Therefore, we propose shifting the focus of research from the pursuit of an ideal target forward to the development of multiple distinct CAR coding constructs, enabling the targeting of each pathological cell within a heterogeneous blast cell population.

Among the considered AML-associated antigens, we can highlight those that belong to the family of growth factor and cytokine receptors (CD116, CD117, CD123, and CD135). The activation of these receptors triggers intracellular signaling pathways that are vital for survival and proliferation, thereby sustaining the uncontrollable blast cell growth. Therefore, targeting the cytokine and growth factor receptors is perhaps a promising approach to the new CAR coding constructs design.

Targeting NKG2D ligands via the NKG2D fragment as a recognizing domain of CAR introduced an elegant way of targeting cell receptors [119]. The NKG2D fragment may recognize all the possible NKG2D ligands. Then, there is no need to create a group of scFv sequences, each specific to an NKG2D ligand. Designing a CAR specific to growth factor receptors using the sequences of their natural ligands may enable high affinity and specificity of the target recognition. The concept has already been proved in a number of research studies [66,120,121,122]. Creating the array of CAR variants specific to different molecular targets can be streamlined by selecting the natural ligands as the CAR recognizing domains. 

## Figures and Tables

**Table 1:** Potential molecular targets for CAR-T cells in AML therapy. AML; acute myeloid leukemia. CD; cluster of differentiation. NK; natural killer. HSC; hematopoietic stem cells.

Antigen	Frequency of Expression on the Blast Cells in AML	Presence on All the Blast Cells	Potential on-Target Off-Tumor Toxicity	Biological Function in AML
CD7	30% of cases		T cells, NK cells	Metastasis
CD33	85–90% of cases		Neurotoxicity	
CD38	50–90% of cases	Antigen density may vary (300–6000 protein/cell) [118]	Diverse	Migration
CD44v6	60% of cases		Diverse	Homing, migration, and proliferation
CD70	34–39% of cases	No	Monocytes	Blast cells’ stemness
ILT3 (CD85k)	Mostly M4 and M5 AML		Dendritic cells, monocytes, osteoclasts, endothelial cells	Malignant cell infiltration and evading immune surveillance
CD116	63–78% of cases (mostly M4 and M5 AML)	No	Granulocytic and monocytic lineage, myeloblasts	Leukocyte expansion, proliferation, survival
CD117	80–90% of cases	No	HSC	Proliferation, survival
CD123	70–80% of cases	No	Myeloid progenitor cells	Proliferation, survival
Flt3 (CD135)	>80% of cases		Dendritic cells	Proliferation, survival
B7-H3 (CD276)	37% of cases			Proliferation, migration, and inhibiting tumor microenvironment
Siglec-6 (CD327)	60% of cases	No	B cells, mast cells, placenta	
TIM-3 (CD366)		No	T-cell subtypes, macrophages	Inhibiting tumor microenvironment
CLL-1 (CD371)	>80% of cases	No	Granulocytic and monocytic lineage	
FRb	70% of cases		Monocytic lineage	Leukocyte expansion
NKG2D Ligands	75% of cases		Damaged or infected cells	Evading immune surveillance
